# Less invasive coronary artery revascularization with a minimized extracorporeal circulation system: preliminary results of a comparative study with off-pump-procedures

**DOI:** 10.1186/1749-8090-8-75

**Published:** 2013-04-11

**Authors:** Thorsten Wittwer, Anton Sabashnikov, Parwis B Rahmanian, Yeong-Hoon Choi, Mohamed Zeriouh, Thorsten O Mehler, Thorsten Wahlers

**Affiliations:** 1 Department of Cardiothoracic Surgery, Heart Center, University Hospital of Cologne, Kerpener Str. 62, Cologne, 50924, Germany; 2 Department of Anesthesiology, University Hospital of Cologne, Kerpener Str. 62, Cologne, 50924, Germany

**Keywords:** Minimized extracorporeal circulation, Coronary revascularization, Less-invasive cardiac surgery, Off-pump-surgery

## Abstract

**Background:**

Coronary-artery-bypass-grafting (CABG) with conventional extracorporeal circulation (CECC) is associated with adverse effects such as systemic inflammatory response leading to a decrease in systemic vascular resistance and hemodynamic instability. Modern "less invasive" procedures have been established recently which potentially avoid negative side effects of CECC. The aim of this study was to compare perioperative outcome following coronary revascularization using either a minimized extracorporeal circuit (Mini-HLM) or off-pump technique (OPCAB).

**Methods:**

In this prospective ethics-approved trial, 120 patients referred for CABG were randomly assigned either to off-pump coronary artery bypass (OPCAB) or to a Mini-HLM procedure. Patient demographics, preoperative characteristics and extensive postoperative outcome were analyzed for both groups. Hemodynamic data were measured at seven time points perioperatively.

**Results:**

Operation-time was longer in the Mini-HLM group (178,3 ± 32,9 min) compared to OPCAB (133,2 ± 32,7 min, p < 0,001) with higher graft numbers in Mini-HLM group (3,11 ± 0,7 vs. 1,78 ± 0,7, p < 0.001). There were no significant differences in perioperative hemodynamic criteria, catecholaminergic support, hospital (p = 0,534) and intensive care unit stay (p = 0,880), ventilation time (p = 0,113), blood loss (p = 0,570), transfusion requirements, postoperative atrial fibrillation rate (p = 0,706) and neurocognitive disturbance (p = 0,297). No deaths and no myocardial infarctions were observed.

**Conclusions:**

Coronary revascularisation with Mini-HLM represents a suitable and "less invasive" procedure which achieves all benefits of OPCAB but may allow for less demanding revascularisation than OPCAB in special patients with complex coronary anatomy and can therefore be used both on a routine basis and in all "conversion" cases of OPCAB.

## Background

Coronary artery bypass grafting (CABG) using conventional cardiopulmonary bypass (CCPB) was first introduced into clinical practice in 1950 [1] and is currently the most common procedure in surgical treatment of multivessel coronary artery disease (CAD) [2-5]. Although several technical as well as surgical and anesthesiological improvements have been conducted [6], the traditional approach requires the use of extracorporeal circulation (ECC), total aortic cross-clamping and myocardial ischemia. In the last two decades, there has been significant interest in the development of alternative surgical treatment procedures in order to minimize the adverse effects associated with the use of CCPB [7,8]. The relevance of this trend has received considerable attention mainly with regard to minimizing the contact of blood with foreign surfaces [9] and thus reducing or completely avoiding the cardiopulmonary bypass. As a result, there are several less-invasive surgical options for coronary revascularization: either with the use of a minimized extracorporeal circuit (Mini-HLM), in off-pump technique via median sternotomy (OPCAB) or via anterolateral mini-thoracotomy in terms of the MIDCAB (minimally invasive direct coronary artery bypass) approach to the culprit LAD lesion with or without hybrid PTCA of additional coronary lesions [10,11]. Currently, advantages and disadvantages of different approaches are intensively discussed. Consequently, many recent studies have focused on the benefits and drawbacks of different techniques. In comparison to other methods usually only one coronary vessel can be revascularized using the MIDCAB technique [12]. Even though the early control angiography results confirmed a very good global MIDCAB patency rate (97.8%) and appear to be even superior to the corresponding results of conventional bypass surgery [11,13], the application of MIDCAB in multivessel disease has been a subject of controversy regarding the completeness of myocardial revascularization. The objective of this paper is to critically examine both two alternative procedures for elective treatment of multivessel CAD in less invasive coronary artery bypass surgery: OPCAB and Mini-HLM. Our preliminary investigation was conducted with special attention to the evaluation of potential differences between both CABG procedures with regard to perioperative outcome including invasive serial hemodynamic characteristics.

## Methods

In this prospective randomized clinical trial, 120 patients referred for isolated elective CABG between December 2008 and July 2011 were enrolled in the study and preoperatively randomized either to OPCAB (n = 44) or Mini-HLM (n = 76) group. The study was performed with the approval of the ethics committee of the medical faculty of the University of Cologne and was in compliance with the Helsinki declaration. For randomization, we initially decided to use the patient chart number with even last digits for the OPCAP- and uneven last digits for the mini-HLM cohort. Unfortunately, this method resulted in an asymmetric distribution of patients rather than a 1:1 ratio; however, in order to avoid a change in the randomization procedure in the ongoing study we continued with this method. After assessment of each coronary angiographic examination by the operating surgeon with special emphasis on the general acceptability all patients were informed about the study. The appropriate written informed consent was obtained from each patient of this clinical trial, and all perioperative data were collected electronically. Included were patients with coronary artery disease and indication for CABG. In order to guarantee the comparability of the two study groups, patients with the following conditions were excluded from the trial: significantly impaired left ventricular pump function (ejection fraction <20%), preoperative catecholaminergic support, hemodynamic instability, mechanical circulatory assistance as well as urgent and emergent cases.

The primary end point of our clinical trial was the in-hospital mortality, secondary end points were intra- and postoperative characteristics with the emphasis on invasive hemodynamic assessment using a pulmonary artery catheter (PAC) as described below.

### Surgical procedure and perioperative care

All operations were performed by only three senior cardiac surgeons with high experience in OPCAB revascularization in cooperation with experienced anesthesiologists and perfusionists in our department. The surgical access was performed via median sternotomy. The left (LIMA) and - when applicable - right internal mammary artery (RIMA) were harvested together with veins and surrounding tissue as a pedicle graft in both groups. Additional graft material (saphenous vein) was harvested using minimal invasive technique. Body temperature was kept as normal as possible, either by the heat exchange system of the Mini-HLM or by specific warming blankets of different manufacturers in the OPCAB group. No patient was discharged from OR with a temperature below 36.0°C. In all cases of CABG using Mini-HLM several doses of Calafiore warm blood cardioplegia were given in an antegrade fashion through the aortic root after establishment of cardiopulmonary bypass with an arterial cannula in the ascending aorta and a two-stage venous cannula introduced through the right atrium. In OPCAB, a Medtronic Urchin Evo Heart Positioner (Medtronic, Inc., Minneapolis, USA) was used to allow for effective positioning of the heart and enhanced visualization of the anastomotic sites during beating heart surgery. A Medtronic Octopus System was utilized to enhance visibility at the anastomotic site.

Postoperatively, all patients were left intubated and were transferred to the cardiac ICU. Perioperative care of the patients was performed according to standard protocols of our department.

### Technical characteristics of Mini-HLM

The ROCsafe Mini-HLM is a closed loop minimized circulatory circuit that consists of a centrifugal pump head (Sarns™ 164275X, Terumo Cardiovascular Systems, Ann Arbor, MI, USA), a microporous polypropylene fiber oxygenator (CAPIOX® RX15E, Terumo Corporation, Tokyo, Japan) including a heat exchange system and a 40-μm polyester arterial blood filter (AL8X, Pall, East Hills, NY, USA). The low prime oxygenator of ROCsafe Mini-HLM facilitates high oxygen transfer (oxygen transfer capacity: 337 mL/min at 5 L/min blood flow) in combination with low priming volume (oxygenator priming volume: 135 mL). Additionally this model provides an air removal function on the venous line allowing an effective air elimination before it enters the centrifugal pump. In case of air in the venous line, the ultrasound-controlled bubble detector leads to a speed reduction of the centrifugal pump, closure of the electronic venous line occluder (Terumo Corporation, Tokyo, Japan) and removing of trapped air from the bubble trap (CAPIOX® BT15X, Terumo) via vacuum suction device. Residual micro air can be eliminated by the oxygenator and arterial filter. This process takes a few seconds before circulation can be resumed. The total component priming volume is approximately 500 ml (Table 1).

**Table 1:** Technical characteristics of Mini-HLM, ROCsafe

**Module**	**Technical characteristics**
**Oxygenator**	
Type	CAPIOX® RX15E
Priming volume (static)	135 mL
Membrane surface area	approx. 1,5 m2
Fiber material	microporous polypropylene
**Bubble trap**	
Type	CAPIOX®BT15X
Priming volume	150 mL
Filter pore area	60%
Filter pore size	170 μ
Filter material	Polyester
**Arterial filter**	
Type	Terumo® AL8X
Priming volume	170 mL
Filter area	0,063 m^2^
Filter pore size	40 μ
Filter material	Polyester
**Centrifugal pump head**	
Type	SarnsTM 164275X
Priming volume	48 mL
Surface area	0,02 m^2^
**Total component priming volume**	**503 mL**

### Assessment of patient’s hemodynamics and clinical parameters

Measurement of perioperative hemodynamic parameters was routinely performed using Swan-Ganz-CCOmbo PAC (Edwards Lifesciences), which was inserted in Seldinger technique during the induction of anesthesia and removed directly after the last measurement 24 hours postoperatively in the ICU. Systemic vascular resistance (SVR), pulmonary vascular resistance (PVR), cardiac output (CO), cardiac index (CI), pulmonary capillary wedge pressure (PCWP), central-venous pressure (CVP), mixed venous oxygen saturation (svO2), arterial systolic (ASP), diastolic (ADP) and mean pressures (AMP) as well as pulmonary arterial systolic (PASP), diastolic (PADP) and mean pressures (PAMP) were recorded at seven time points perioperatively: skin cut ("OP-0"), post Mini-HLM/OPCAB-stabilizer ("OP-1"), skin closure ("OP-2"), one hour ("ICU-1"), six hours ("ICU-2"), 12 hours ("ICU-3") and 24 hours after surgery ("ICU-4") respectively. These data were collected automatically using clinical pressure monitoring equipment in combination with the Vigilance Monitor without performing bolus thermodilution measurements. SvO2 was monitored by fiberoptic reflectance spectrophotometry available in the PAC model 744HF75 that was used in our study. Additionally this model provides an antimicrobial heparin coating in order to minimize viable microbe count of the catheter surface during placement as well as to guarantee a long-term protection against thrombogenesis [14].

Perioperative clinical characteristics were assessed by evaluating the medical charts of the patients during their hospital stay and after their discharge. The data of the perioperative hemodynamic measurements were directly documented at the appropriate time points.

### Statistical analysis

Statistical analysis was performed by SPSS 19.0 (SPSS Inc., Chicago, IL, USA). All continuous variables are expressed as mean ± standard deviation (SD). Categorical data are expressed as frequencies and percentages. Statistical analysis of metric and dichotomous parameters was carried out using T test and Chi-square test respectively. A p < 0,05 was considered statistically significant.

## Results

The preoperative data were comparable between patients of both groups of the study. Table 2 represents basic demographic data, patient comorbidities and general clinical characteristics of all patients undergoing either OPCAB or CABG using Mini-HLM. The mean age of the population was 65,6 ± 10,8 years within a range of 40–89 years. 18,3% (n = 22) of patients were female. A history of hypertension, hyperlipidemia, diabetes, previous stent implantation, statin therapy, atrial fibrillation, myocardial infarction as well as logisticEuroscore and preoperatively assessed heart pump function were equally distributed between groups. There were no considerable discrepancies concerning serological parameters (creatine kinase (CK), CK-MB, Troponin T, hemoglobin, creatinine and hematocrit) as well.

**Table 2:** Patient´s demographics and perioperative data

	**Mini-HLM**	**OPCAB**	**Total**	**p-value**
Age (yrs)	65,8 ± 11,2	65,2 ± 10,4	65,6 ± 10,8	0,79
Female	9 (12,2%)	13 (28,3%)	22 (18,3%)	**0,032**
Height (cm)	173,5 ± 7,8	172,3 ± 8,2	173 ± 7,9	0,448
Weight (kg)	84,5 ± 13	82,8 ± 13,6	83,9 ± 13,1	0,506
Euroscore	3,2 ± 2,3	3,2 ± 2,3	3,2 ± 2,3	0,938
Vessel disease	2,7 ± 0,5	2,13 ± 0,9	2,1 ± 0,9	0,65
Preoperative creatinine (mg/dl)	0,9 ± 0,2	0,9 ± 0,3	0,9 ± 0,2	0,879
Preoperative haemoglobin (g/dl)	12,6 ± 1,7	12,4 ± 1,7	12,5 ± 1,7	0,557
Preoperative CK (U/l)	75,1 ± 59,2	79,5 ± 46,8	76,8 ± 54,6	0,675
Preoperative CK-MB (U/l)	9,5 ± 4,1	9,9 ± 5,2	9,7 ± 4,6	0,546
Preoperative Troponin T (ug/l)	0,02 ± 0,06	0,03 ± 0,1	0,02 ± 0,08	0,62
Preoperative haematocrit (%)	36,8 ± 6,2	36,3 ± 4,5	36,6 ± 5,6	0,658
Preoperative EF (%)	61,9 ± 14,7	57,8 ± 15,3	60,7 ± 14,9	0,271
Preoperative LVEDP (mmHg)	15,2 ± 7,7	13,6 ± 10,8	14,6 ± 8,8	0,498
Diabetes mellitus	13 (17,6%)	10 (21,7%)	23 (19,2%)	0,636
Arterial hypertonia	58 (78,4%)	32 (69,6%)	90 (75,0%)	0,298
Hyperlipoproteinemia	57 (77,0%)	32 (69,6%)	89 (74,2%)	0,396
Previous stent implantation	14 (18,9%)	12 (26,1%)	26 (21,7%)	0,371
Previous statin therapy	63 (85,1%)	36 (78,3%)	99 (82,5%)	0,38
Preoperative atrial fibrillation	4 (5,4%)	0	4 (3,3%)	0,297
History of acute myocardial infarction	29 (39,2%)	12 (26,1%)	41 (34,2%)	0,168
Prior bypass surgery	0	0	0	
Previous lysis of intravascular thrombus	0	0	0	
Preoperative hemodynamic instability	0	0	0	

*CK* creatine kinase, CK-MB creatine kinase isoenzyme, *EF* ejection fraction, *LVEDP* left ventricular end-diastolic pressure.

**Figure 1:**
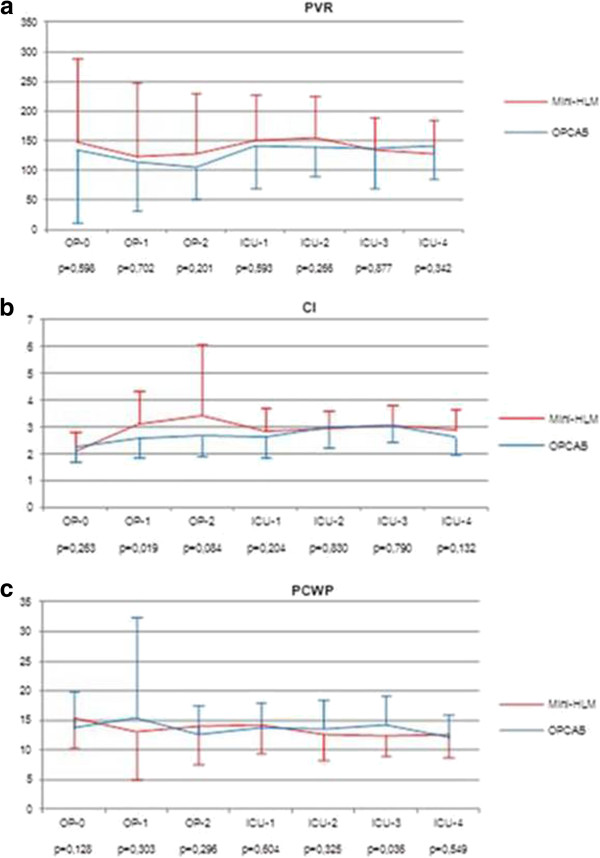
**Perioperative hemodynamic measurements using a pulmonary artery catheter (Edwards Lifesciences).** There are no significant differences between the two groups at most time points (p > 0,05), few differences (p < 0,05) have no clinical significance because the corresponding values remain in the normal range. **a**: Pulmonary vascular resistance (PVR), y-axis in [dyne*sec/cm^5^]; **b**: Cardiac index (CI), y-axis in [l/min/m^2^ BSA]; **c**: Pulmonary capillary wedge pressure (PCWP), y-axis in [mmHg]. "OP-0", skin cut; "OP-1", post Mini-HLM/OPCAB-stabilizer; "OP-2", skin closure; “ICU-1”: one-hour post-op; "ICU-2": six-hour post-op; "ICU-3”: 12-hour post-op; "ICU-4": 24-hour post-op.

Every single patient in both the Mini-HLM and the OPCAB group received a LIMA-graft to the left anterior descending artery (LAD) as a significant LAD-stenosis/occlusion was present in all operated patients. The usage of bilateral internal mammary artery was comparable between the two groups (Table 3). In the OPCAB group, a significantly lower mean number of grafts was performed as compared to the Mini-HLM group (1,78 ± 0,7 vs. 3,11 ± 0,7, p < 0.001) although anatomically complete revascularization was intended in all patients of both treatment cohorts. By surgical expertise of the operating surgeons, inappropriate incomplete revascularization was precluded in each patient. The procedure time was significantly longer in the Mini-HLM group (178,3 ± 32,9 min) compared to the OPCAB group (133,2 ± 32,7 min, p < 0,001). However, there were no significant differences in perioperative hemodynamics (Figure 1a-c), inotropic support, duration of hospital (p = 0,534) and intensive care unit stay (p = 0,88), ventilation time (p = 0,113), blood loss (p = 0,57), transfusion requirements, postoperative atrial fibrillation rate (p = 0,706) and neurocognitive disturbance (p = 0,297). There were no deaths and no myocardial infarctions observed in either group. All cases were performed as intended with no need for conversion to CCPB (Tables 3 and 4).

**Table 3:** Intraoperative data

	**Mini-HLM**	**OPCAB**	**Total**	**p-Wert**
Number of distal anastomoses	3,11 ± 0,7	1,78 ± 0,7	2,6 ± 0,97	**<0,001**
Operating time (min)	178,3 ± 32,9	133,2 ± 32,7	160,9	**<0,001**
LIMA and RIMA usage	19 (25,7%)	9 (19,6%)	28 (23,3%)	0,51
Transfusion of PRBC	0,4 ± 0,9	0,4 ± 1,0	0,42 ± 1,0	0,97
Transfusion of FFP	0,4 ± 1,3	0,1 ± 0,5	0,3 ± 1,1	0,16
Transfusion of platelets	0,2 ± 0,5	0,1 ± 0,3	0,2 ± 0,4	0,19
Bypass time (min)	75,9 ± 18,6			
Cross-clamp time (min)	41,1 ± 11,6			
Reperfusion time (min)	26,2 ± 8,4			

*FFP* fresh frozen plasma, *LIMA* left internal mammary artery, *PRBC* packed red blood cells, *RIMA* right internal mammary artery.

**Table 4:** Postoperative data

	**Mini-HLM**	**OPCAB**	**Total**	**p-Wert**
Ventilation time (min)	15,4 ± 7,9	13,4 ± 3,8	14,6 ± 6,7	0,113
Total chest tube drainage (ml)	1266 ± 707	1039 ± 449	1178 ± 628	0,057
ICU stay (d)	2,6 ± 1,3	2,6 ± 2,3	2,6 ± 1,7	0,880
In-hospital stay (d)	12,5 ± 7,1	11,5 ± 9,3	12,1 ± 8,0	0,534
Transfusion of PRBC	1,4 ± 1,9	0,8 ± 1,2	1,2 ± 1,7	0,090
Transfusion of FFP	0,74 ± 1,9	0,6 ± 1,3	0,7 ± 1,7	0,651
Transfusion of platetets	0,3 ± 0,6	0,1 ± 0,4	0,2 ± 0,5	0,270
Catecholaminergic support				
Norepinephrine	3,7 ± 3,9	12,3 ± 58,5	7,0 ± 36,3	0,218
Epinephrine	0	1,3 ± 7,9	0,5 ± 4,9	0,161
Dobutamine	288 ± 327	195 ± 370	253 ± 346	0,161
Milrinone	0	0,8 ± 5,3	0,3 ± 3,3	0,210
Symptomatic transitory psychotic syndrome	4 (5,4%)	0	4 (3,3%)	0,297
Cerebral vascular accident	2 (2,7%)	0	2 (1,7%)	0,523
Atrial fibrillation	30 (41,7%)	21 (45,7%)	51 (43,2%)	0,706
Use of class III antiarrhythmics	15 (20,5%)	7 (15,2%)	22 (18,5%)	0,629
Readmission to the ICU	3 (4,1%)	1 (2,2%)	4 (3,3%)	1,0
Reintubation	1 (1,4%)	1 (2,2%)	2 (1,7%)	1,0
Sepsis	1 (1,4%)	1 (2,2%)	2 (1,7%)	1,0
Creatinine 24–48 hours after surgery (mg/dl)	1,15 ± 0,38	1,06 ± 0,37	1,12 ± 0,38	0,202
CVVH	0	0	0	
Deep sternal wound infection	6 (8,1%)	2 (4,3%)	8 (6,7%)	0,709
Sternal instability	3 (4,1%)	1 (2,2%)	4 (3,3%)	1,0
Hospital mortality	0	0	0	
Perioperative myocardial infarction	0	0	0	
CPR	0	0	0	

*CPR* cardiopulmonary resuscitation, *CVVH* continuous veno-venous hemodialysis, *FFP* fresh frozen plasma, *ICU* intensive care unit, *PRBC* packed red blood cells.

Serial assessment of patient’s hemodynamics did not result in statistically significant differences in most instances between the two groups (Figure 1, a-c). Despite the existence of minor significant differences, the corresponding values were within the normal range in both groups, indicating no major clinical relevance.

## Discussion

CABG is the treatment of choice in patients with three-vessel CAD as recommended by recent Guidelines [15]. A considerable number of reports have been published on CABG using ECC and proved that it is a safe and effective treatment of multivessel CAD. However, the standard approach via median sternotomy with the use of cardiopulmonary bypass (CPB) and aortic cross-clamping is associated with certain negative side effects such as systemic inflammatory response syndrome (SIRS) which leads to potentially increased mortality and morbidity [2,10,16-18].

To avoid these adverse effects, alternative approaches such as OPCAB, MIDCAB and interdisciplinary hybrid procedures have been introduced into clinical practice. However, these approaches are sometimes technically demanding and therefore do not always represent the best available technique in specific patients. Particularly off-pump procedures require sufficient exposition of target vessels which can be very demanding particularly when marginal branches need to be revascularized which may result in severe hemodynamic instability due to cardiac displacement. Furthermore, diffuse CAD with severe calcified target vessels may not always be suitable for revascularization during beating heart surgery. In these instances, the utilization of minimized extracorporeal circuits may offer benefits for these patients as these Mini-HLM systems have been proposed to avoid the potentially harmful effects of CCPB. The basic idea of Mini-HLM is to ensure adequate perfusion by a closed, extremely minimized circuit based on a rotary blood pump and a high-performance membrane oxygenator with elimination of blood-to-air contact by avoiding a venous reservoir, minimizing hemodilution and mechanical blood trauma and significant reduction of contact activation by reduced foreign surfaces [19]. Meanwhile, a clear superiority of Mini-HLM systems could be proven when compared to conventional CPB circuits [7,9,10,16,20].

In the present study we compared OPCAB revascularization with on-pump procedures utilizing a Mini-HLM, and comparable excellent clinical results could be demonstrated in both groups without any significant differences. Particularly mortality, postoperative morbidities and complication rates as well as clinical variables of the perioperative period, e.g., blood loss, transfusion requirements, ventilation- and total-hospital-times were not significantly different between both groups. Nevertheless, this is an extremely important result with high clinical impact as the use of CCPB has been associated with a couple of the above-mentioned negative side effects. Based on these concerns with CCPB, a significant amount of studies deal with the OPCAB procedure as as a well-established and safe minimal invasive procedure for coronary artery revascularization [21,22]. This technique has been extensively studied and was shown to reduce the incidence of post-operative complications such as SIRS, myocardial and cerebral dysfunction and hemodynamic instability associated with the use of CCPB [18]. Additionally the lower cost factor and availability of mechanical stabilizers [22,23] made this approach more appealing, even though this method of myocardial revascularization appeared to be technically more demanding and more complex and although some authors report even higher hospital costs with longer hospital stays after OPCAB procedures [5]. Further findings strongly support the general impression that OPCAB represents an alternative approach for patients with increased risk of ECC on the basis of lower mortality [23,24]. For a long time this method was the only alternative surgical approach for patients with severe comorbidities, who had absolute contraindications for operations using ECC [25]. Among numerous other benefits, improved perioperative outcome including reduced catecholaminergic support, transfusion requirements, ventilation time and lower impairment rate of the preexistent renal insufficiency was shown in patients with at least 50% main left coronary artery stenosis operated on in OPCAB technique as compared to a CCPB group [26].

In our study the utilization of Mini-HLM, however, resulted in a completely comparable low complication rate compared to the off-pump group. Therefore, broader introduction of minimized circulatory systems into clinical practice has the promising potential to reduce negative side effects of standard CPB systems. Moreover, hemodynamic measurements in the present study did not show any significant differences in the postoperative course between the two groups suggesting that the use of Mini-HLM is safe and did not lead to hemodynamic instability.

A significant finding of our study was a higher rate of distal anastomoses in the Mini-HLM group. As an often quoted limitation of OPCAB procedures, complete coronary revascularization might not be achievable in all patients by off-pump techniques owing to the complex anatomy of coronary lesions and the possibility of hemodynamic instability while the beating heart is manipulated. In a study with 2203 patients undergoing off-pump and on-pump procedures, Shroyer et al. [18] have reported a significantly lower revascularization rate (2,9 ± 0,9 and 3,0 ± 1,0, respectively; p = 0,002) as well as a higher proportion of patients with fewer grafts than originally planned in the off-pump group than in the on-pump group (17.8% vs. 11.1%, P < 0.01). The overall rate of graft patency was lower in the off-pump group than in the on-pump group (82.6% vs. 87.8%, P < 0.01). However, the required level of surgical experience in OPCAB revascularization was not well determined in this ROOBY trial which is considered to be a certain drawback of that study. Nevertheless, other reports have also shown a higher number of grafts [10,21,22] and a lower rate of repeat revascularization [27] observed in on-pump patients as compared to OPCAB-operated patients. Correspondingly, in the study by Alamanni et al., OPCAB was identified as the most significant predictor (OR 7,1, 95% CI 5,9-8,3, p < 0,001) of incomplete revascularization [28]. Additionally, a study comparing standard CPB, minimized extracorporeal circulation system (MECC) and OPCAB (10) has shown a similar revascularization rate in the on-pump groups (3,2 ± 0,6 in the MECC group and 3,4 ± 0,7 in the CCPB group) while the revascularization rate was significantly lower in the off-pump group (1,9 ± 0,8, p = 0,01). Similarly, in our study the overall bypass number was lower in the OPCAB group which was mainly due to unexpected intraoperatively observed multiple, complex and diffuse disease or small vessels unsuitable for surgical revascularization.

However, in the available literature there is a substantial uncertainty on the definition and evaluation of the “adequacy” of myocardial revascularization, and numerous inconsistent definitions for “complete (CR)” and “incomplete (IR)” revascularization account for some controversies regarding mid- and long-term event-free survival [29]. Generally, a coronary revascularization is considered to be “anatomically complete” when all vessels with clinically significant stenoses (vessel diameter > 1.5 mm; stenosis > 50%) are treated, irrespective of the underlying myocardial function, whereas a “functionally complete” revascularization refers to situations in which only lesions supplying a viable myocardium are treated [29]. Myocardial revascularization may therefore be both anatomically incomplete but still functional adequate [30]. According to Mohr et al. [31], even when CR is still the major goal of surgical revascularization, there remain several reasons to perform “reasonable” surgical IR, based on either preoperative assessment (e.g., nondominant diseased right coronary artery, non-vital myocardium, attempt to avoid cardiac arrest for off-pump procedures in high-risk patients, limited graft material), or more unexpected findings intraoperatively like in our series (i.e., small target vessels with severe calcifications, intramyocardial coronaries, coronary injury). Interestingly, in this important study on 8806 coronary patients with multivessel disease, IR of the circumflex or right coronary artery - in the presence of a LIMA bypass grafted to the LAD in all cases - was not associated with any detrimental effects on 1- or 3-year survival in comparison to those patients with CR [31].

Therefore, due to these difficulties in definition and impact of the “completeness of revascularization” and as each patient of our two cohorts did receive a LIMA bypass to the LAD, it was not the primary goal of this preliminary study to specifically focus on complete or incomplete revascularization rates but rather to evaluate the immediate perioperative clinical outcome including intensive care data and hemodynamic assessment. Nevertheless, anatomically complete revascularization was intended in all Mini-HLM- and OPCAB-cases of our series, and it was guaranteed by surgical expertise that “inappropriate incomplete revascularization” as recently described by Taggart [32] was avoided in each single patient. There is no doubt, however, that the revascularization rate particularly in off pump patients depends on optimal surgical experience. On the other hand, several factors such as diffuse calcification, peripheral stenosis and intramyocardial localization of the coronary arteries may complicate the procedure. Logically, the question always rises as to whether a lower number of grafts in myocardial revascularization with or without the use of CPB may be associated with technical or anatomical difficulties, and thus represents a risk of incomplete myocardial revascularization with potentially inferior outcome. Even if incomplete revascularization might be unavoidable in some specific instances, is was shown in a recent subgroup analysis of the well-respected SYNTAX data [33] that in surgically treated patients adverse events in terms of MACCE and need for repeat revascularization are not significantly increased following IR as compared to CR.

Our study has revealed comparable clinical and hemodynamic parameters between the two groups even though the operating time was significantly longer in the Mini-HLM group as compared to the OPCAB group (Tables 2 and 3). The reason for this result appears to be less systemic inflammatory reaction and organ damage due to reduced priming volume and total surface area of the circuit as well as less activation of platelets and granulocytes compared to CCPB as previously reported [2,8,10]. Other advantages of Mini-HLM over CCPB such as reduced hemodilution, a decreased inflammatory response in terms of interleukin-6 (IL-6) and C3a values as well as improved clinical outcome were also shown in high-risk CABG patients [34]. Our general findings are also consistent with those of Mazzei et al. [8] who found that the release of inflammatory markers, as well as length of intensive care unit, hospital stay, complication rate and need for allogenic transfusion were similar in the OPCAB and a Mini-HLM group. Additionally, it was shown that use of modern Mini-HLM systems as compared to CCPB is associated with significantly reduced release of circulating endothelial cells (CEC) who are considered to represent a novel and hypersensitive marker of the severe intrinsic endothelial damage with detachment of endothelial cells into the blood stream caused by CCPB [35]. Recently, CEC release in Mini-HLM procedures was found to be as low as it could be shown for OPCAB procedures.

For the first time internationally, in this investigation perioperative hemodynamic parameters were directly compared in order to appraise the extent of systemic inflammation induced by surgical technique with may lead to potential adverse effects such as deterioration of hemodynamics. The results of this study did not show any significant differences in the perioperative hemodynamics between both groups in spite of total aortic cross-clamping and cardiac arrest in Mini-HLM operations. Furthermore, similar amounts of catecholamines were used in both groups. A possible explanation of our favourable results may be the numerous conceptional advantages of Mini-HLM, resulting in less perioperative systemic inflammation and hemodynamics comparable to off-pump procedures. Our findings which showed that there were no significant differences between the two groups at most time points are also in accordance with clinical observations. The few statistical differences have no clinical significance because the corresponding values remain within the normal range.

This preliminary study has a number of certain limitations. First, it was a single center study with a rather small sample size. Therefore caution must be applied, as the findings might not be transferable to larger cohorts. Furthermore, clinical outcome analysis focused initially on short-term peri- and postoperative observations, and no long-term results are provided yet. However, ongoing experience with this continuous ethics-approved study will result in specific intermediate- and long-term evaluations of bypass function and persistent relief or recurrence of ischemic symptoms.

## Conclusion

Based on the results of this study, coronary revascularization by use of the ROCsafe™ -Mini-HLM system represents a suitable and "less invasive" procedure which achieves all benefits of OPCAB but may allow for less demanding revascularization than OPCAB in special patients with complex coronary anatomy. In these patients the use of Mini-HLM and circulatory arrest might improve the surgical approach to the target vessels resulting in potentially improved quality of anastomoses, while retaining the benefits of off-pump procedures compared to standard HLM such as comparable incidence of atrial fibrillation, ventilation time, postoperative bleeding, transfusion requirements, catecholaminergic support etc. as shown in our study. This promising technique can therefore be used both on a routine basis and especially in all patients who are considered to be no optimal candidates for OPCAB.

## Competing interests

The authors declare that they have no competing interests.

## Authors’ contributions

TW: concept of the study, senior cardiac surgeon, extensive revision + final approval of manuscript. AS: Acquisition of data, drafting of manuscript, statistical analysis. PBR: Statistical analysis, revision of manuscript. Y-HC: Concept of the study, revision of manuscript. MZ: Acquisition of data, statistical analysis. TOM: Concept of the study, revision of manuscript. TW: Final approval of manuscript. All authors read and approved the final manuscript.
